# Does Kidney Transplantation With Deceased or Living Donor Affect Graft Survival?

**DOI:** 10.5812/numonthly.12182

**Published:** 2014-07-05

**Authors:** Eghlim Nemati, Behzad Einollahi, Mahboob Lesan Pezeshki, Vahid Porfarziani, Mohamad Reza Fattahi

**Affiliations:** 1 Nephrology and Urology Research Center, Baqiyatallah University of Medical Sciences, Tehran, IR Iran; 2 Department of Nephrology, Imam Khomeini Hospital, Tehran University of Medical Science, Tehran, IR Iran

**Keywords:** Living Donor, Donor, Survival, Kidney Transplantations

## Abstract

**Background:**

There are growing numbers of patients with end-stage renal disease globally at an unexpected rate. Today, the most serious challenge in transplantation is organ shortage; hence, using deceased donor is increasingly encouraged.

**Objectives:**

The aim of the study was to investigate the differences in survival rates between kidney transplant recipients with deceased donor and living donor.

**Patients and Methods:**

In a retrospective cohort study, 218 patients who had undergone kidney transplantation in our institute from April 2008 to September 2010 were recruited. Demographics and post-transplantation follow-up data including immunosuppression regimens, rejection episodes, and survival rates were evaluated. The patients were assigned to two groups according to the donor kidney transplantation: group I, living donor kidney transplants; and group II, deceased donor kidney transplants.

**Results:**

Although there were no significant differences in one-year survival rates of patient and graft between study groups, three-years survival rates of patient and graft were significantly longer in living donor kidney transplants in comparison with the deceased donor kidney recipients (P = 0.006 and P = 0.004, respectively). In Cox-regression model after adjusting for other confounding factors such as age, sex, diabetes mellitus, and first- or second-time transplantation, overall patient and graft survivals were also significantly shorter in deceased kidney transplantation than those who received kidney from a living donor (HR, 3.5; 95% CI, 1.2-10.4; and P = 0.02 for patient survival; and HR, 5.4; 95% CI, 1.5-19.5; and P = 0.009 for graft survival).

**Conclusions:**

We found acceptable short-term survival in both groups; however, living donor recipients continue to have better long-term patient and graft survival rates.

## 1. Background

End-stage renal disease (ESRD) is a disabling disease with high mortality rate. It affects a high percentage of the population and its treatment consumes a considerable portion of national health resources (1-3). On the other hand, the number of patients with ESRD requiring renal replacement therapy (RRT) continues to grow globally at an unexpected rate. Although almost 90% of patients with ESRD live in high-income countries, it has been forecasted that by 2030, more than 70% of these patients would be from developing countries with less than 15% of the world economy (4). Over the past decade, RRT rates have increased worldwide (5, 6). The excellent outcomes of kidney transplantation have resulted in an increasing demand for such a treatment (7). Promising results have made renal transplantation the treatment of choice for majority of patients with ESRD (8, 9). Successful renal transplantation is cost-effective and offers advantages over dialysis in terms of survival and quality of life. Today, the biggest challenge in kidney transplantation is organ shortage; hence, using deceased donor is increasingly encouraged. Although the outcome of living donor kidney transplantation (LDKT) is better than that of deceased donor kidney transplantation (DDKT), the number of LDKT done annually was unchanged for years due to the reluctancy of transplant personnel to impose a major and unnecessary operation to a potential donor. However, because of the rapidly growing waiting list for DDKT and the increasingly longer wait, most centers are now advocating LDKT. In the past two years, the number of living donors has increased. Most of this increase followed the recognition of similar outcomes of living unrelated donor recipients to those of living related non-HLA-identical donor recipients (10). The lack of cadaveric donors along with a rapidly growing number of potential recipients has led into implementation of several strategies such as acceptance of older donors to increase the organ pool and reduce the waiting list for kidney transplantation. However, several studies have demonstrated higher incidences of delayed graft function and poor graft outcomes among kidneys harvested from older donors. Donor age showed no effect on allograft survival; however, kidney allografts from older donors have displayed lower first-year as well as long-term renal function (11).

## 2. Objectives

Differences in actuarial graft survival regarding donor sex have been reported for renal, cardiac, and hepatic allografts; however, the statistics for the latter were derived from small series with limited biostatistical power (12). We aimed to investigate differences in survival rate between LDKT and DDKT.

## 3. Patients and Methods

### 3.1. Study Population

In a retrospective Cohort study, we assessed 218 kidney transplant recipients who had undergone transplantation surgery in our institute from April 2008 to September 2010. Demographics and post-transplantation follow-up data including investigations, immunosuppression requirement, rejection episodes, and survival were collected. The patients were assigned to two groups according to the source of kidney: group I, LDKT; and group II, DDKT. Ethical approval of research was confirmed by local ethic committee of university. Inclusion and exclusion criteria such as all patients were first kidney transplantation. Adult recipients included, etc.

### 3.2. Donor Selection

Donors were required to be healthy adult relatives with compatible ABO blood type and negative serum tests for hepatitis B virus, hepatitis C virus, and human immunodeficiency virus. The donors were evaluated using computed tomography angiography.

### 3.3. Definition

Patient survival was defined as the period from transplantation to death. Graft survival was defined as the period from transplantation until the time hemodialysis was required. Diagnosis of rejection was defined as a decline in renal function or clinical suspicion of acute rejection, which should be confirmed by renal graft biopsy.

### 3.4. Clinical Observations

Immunosuppression was based on cyclosporine (CsA) plus mycophenolate mofetil or azathioprine and prednisolone. In most centers, CsA doses given to kidney recipients are administered mostly upon CsA trough levels. CsA levels was assessed at different times and dose was adjusted as local protocol and when necessary. Our therapeutic target for C0 levels ranged from 200 to 300 ng/mL during the first through the third months, from 100 to 250 ng/mL during the fourth through 12^th^ months, and from 100 to 150 ng/mL after one year of transplantation. Therapeutic target levels for C2 ranged from 800 to 1000 ng/mL during the first through the third months after transplantation and from 400 to 600 ng/mL during subsequent months.

### 3.5. Laboratory Test

Laboratory parameters including creatinine (Cr), uric acid, and blood levels of CsA were measured in whole blood samples using the Cobas Mira-Plus analyzer (Roche).

### 3.6. Post-transplantation Follow-up

All patients were followed up at weekly intervals during the first month post-transplantation, fortnightly for the next three months, monthly for the next six months, and at three-month intervals thereafter. On every visit, renal and liver function were monitored and complete blood counts were performed.

### 3.7. Statistical Analysis

The SPSS version 17.0 for Windows (SPSS Inc., Chicago, IL, USA) was used in all the analyses. Quantitative variables were expressed as mean ± standard deviation, while qualitative variables were shown by number and percentage. Categorical variables were compared with the Chi square or Fisher's exact test whereas the analysis of variance (ANOVA) was performed for continuous variables. Cox regression was used to assess the variables that were significantly associated with negative outcomes of kidney transplant recipient survival according to Univariate analysis. Post-transplantation survival was estimated using the Kaplan-Meier method with the log-rank test. We considered a P value of less than 0.05 as statistically significant.

## 4. Results

### 4.1. Demographical Setting

We enrolled 218 kidney transplant recipients including 144 males (66%) and 74 females (34%). No significant difference was seen between two groups in terms of sex (Table 1). There was no statistically significant differences regarding renal allograft function between two groups (P = 0.08). Males were more likely to be anemic in comparison with females (P = 0.02). Recipients in DDKT group were older than those in LDKT (P = 0.01).

**Table 1.** Demographic Data of the Recipients in Both Groups ^a,b^

Variables	Overall (n = 218)	LDKT (n = 115)	DDKT (n = 103)	P Value
**Gender**				0.3
Male	144 (66)	79 (68.7)	65 (63.1)	
Female	74 (34)	36 (31.3)	38 (36.9)	
**Age of Recipient, y (range)**	43 ± 14 (10-76)	41 ± 14 (10-76)	45 ± 14 (17-71)	0.01
**Follow-up, mo**	29 ± 10	30 ± 11	22 ± 7	0.000
**Graft Loss**	14 (6.4)	5 (4.3)	9 (8.7)	0.2
**Mortality Rate**	18 (8.3)	6 (5.2)	12 (11.7)	0.08
**Number of Admissions After Tx**				0.2
None	66.5	72.2	60.2	
1	20.2	18.3	22.3	
2	9.6	7.8	11.7	
3	2.3	1.7	2.9	
4	1.4	0	2.9	
**Last Serum Creatinine, mg/dL**	1.6 ± 1.0	1.4 ± 0.8	1.7 ± 1.1	0.08
**Systolic BP, mm Hg**	127 ± 15	127 ± 16	126 ± 13	0.4
**Diastolic BP, mm Hg**	78 ± 7	77 ± 9	79 ± 5	0.2
**Hb, g/dL**	11.1 ± 1.1	11.0 ± 2.0	11.2 ± 1.8	0.2
**TG, mg/dL**	128 ± 65	130 ± 71	126 ± 59	0.6
**Chol, mg/dL, **	143 ± 43	142 ± 39	145 ± 48	0.6
**Uric acid, mg/dL**	4.8 ± 1.6	4.4 ± 1.5	5.3 ± 1.7	0.000

^a^LDKT, living donor kidney transplantation; DDKT, deceased donor kidney transplantation; BP, blood pressure; Hb, hemoglobin; TG, triglyceride; DM, diabetes mellitus; and HTN, Hypertension.

^b^Data are presented as mean ± SD or No. (%).

### 4.2. Transplant Outcome

Those with DDKT needed more admission to hospital than those with LDKT (67 admissions in 40 cases and 42 admissions in 29 cases, respectively; P = 0.2).

### 4.3. Differences Between Admission and Graft Survival Rates in Living and Deceased Donor

**Figure 1.**
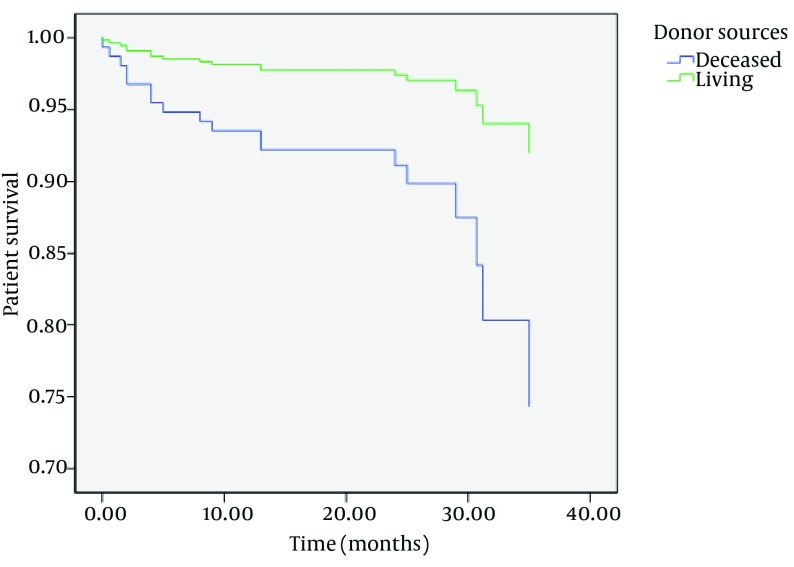
Patient Survival in Two Groups

**Figure 2.**
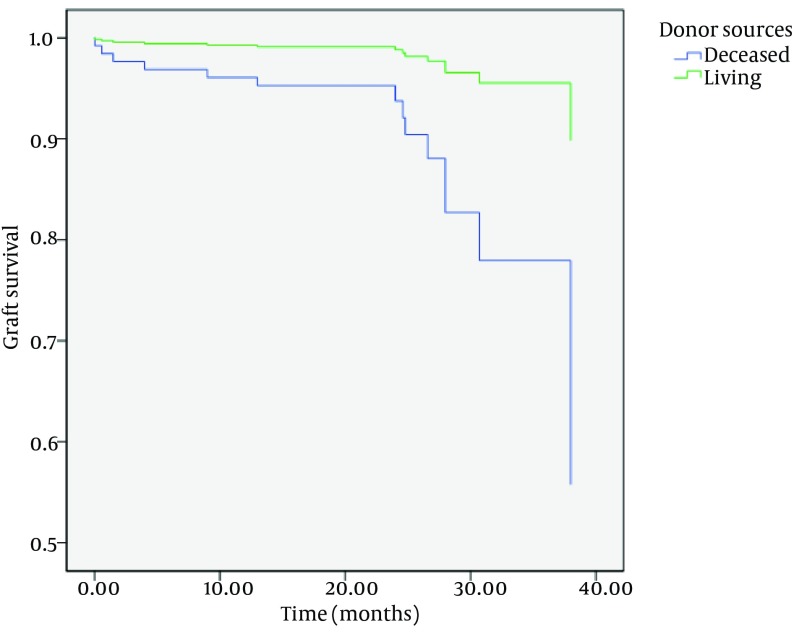
Graft Survival in Two Groups

Although there were no differences between one-year patient and graft survival rates between two groups, there were statistically significant differences in three-year patient and graft survival rates between them (P = 0.006 and P = 0.004, respectively) (Table 2) (Figures 1 and 2).

**Table 2.** Patient and Graft Survival in Two Groups^a,b^

Survival Rates, y	LDKT	DDKT	P Value (Log Rank)
**Graft**			0.004
1	97.4	97.0	
3	96.2	67.4	
**Patient**			0.006
1	95.6	95.1	
3	93.9	45.4	

^a^Abbreviation: LDKT, living donor kidney transplantation; and DDKT, deceased donor kidney transplantation.

^b^Data are presented as %.

### 4.4. Factors Affecting Survival Rates in Kidney Transplant Recipients

After adjusting for other confounding factors such as age, sex, diabetes mellitus, and first or second-time transplantation, patient and graft survivals were significantly shorter in DDKT than LDKT (hazard ratio (HR), 3.5; 95% CI, 1.2-10.4; and P = 0.02 for patient survival; and HR, 5.4; 95% CI, 1.5-19.5; and P = 0.009 for graft survival).

## 5. Discussion

Recent improvements in patient care and immunosuppressive protocols have improved outcome of kidney transplant patients (10). The present study shows that patients with LDKT have better long-term survival than those with DDKT. Feduska et al. revealed that survival rates decreased with increasing in donor’s age (13). Cecka found that the lower graft survival rates were associated with the race, sex, and age, and causes of death of the donor; moreover, early nonfunctional grafts were associated with preservation related factors such as long cold ischemia (14). Matas et al. showed that the result of LDKT has continued to improve; however, donor source affects the outcome in those receiving LDKT (10). Persistent shortage of kidneys for transplantation has forced most transplant centers to obtain and use kidneys from older donors (15). Several studies have demonstrated higher incidences of delayed graft function and poor graft outcomes among kidneys harvested from older donors. Donor age showed no effect on allograft kidneys survival; however, allograft kidneys from older donors displayed lower first-year and long-term renal function (11). Donor age was identified recently as a major factor that determines long-term outcomes; however, the responsible mechanism for increased graft loss of older donor kidneys is unknown. It is hypothesized that increased graft loss of older donor kidneys results from an increased incidence of acute interstitial rejection episodes in the late posttransplantation years. It is proposed that kidneys from older donors are more immunogenic than kidneys from young ones and acute rejection episodes result in functional deterioration. Contrary to interstitial rejection in kidneys from younger donors, kidneys from old donors seem to have an impaired ability to restore tissue (16). Similarly, the result of DDKT in infants and children younger than five years of age has been suboptimal in the past. Reports of the use of children cadaver kidneys for transplantation into children and adult recipients has yielded discrepant results. Fine showed that when cadaver kidneys from donors younger than six years of age were used, there would be the potential for decreased graft survival rates and an increased incidence of technical complications; however, the use of children’s cadaver kidneys can provide adequate graft function in both children and adult recipients and the use of such kidneys should increase the number of kidneys available for transplantation (17). It is shown that damage by atherosclerosis before those microvascular bench reconstructions of the renal artery increases the possibility for safe transplantation of older kidneys without performing a double renal transplantation (18); therefore, atherosclerosis is one of most important reasons for this increased survival in DDKT. In addition, despite matching, early graft function is adversely affected by prolonged cold storage in recipients of younger as well as older donor kidneys (19). Some studies demonstrated that despite a higher degree of HLA mismatching, kidney grafts from living unrelated donors had high survival rates than grafts from cadaver; we think that the crucial difference in survival between living unrelated grafts and cadaveric grafts is that about 10% of the cadaveric grafts are damaged before removal, which is indicated by the 10% difference in graft-survival rates. Once the total nephron mass is compromised, hyperfiltration of the remaining nephrons ultimately leads into graft failure (20, 21); however, this important cause of failure is rarely recognized and instead, the failure is often attributed to chronic rejection (20). The association of the chronic kidney rejection with renal mass was demonstrated in rats; they had a lower rate of chronic rejection when an additional allograft kidney was implanted and had a higher rate when implanted kidneys were reduced in size (21). In addition, there are evidences that demonstrate the effect of brain death (premortem shock and cytokine release), organ preservation, and ischemia-reperfusion injury on the transplantation outcome. The procedure of flushing and keeping the kidney cool during retrieval and storage, either on ice or in a pulsatile perfusion machine while awaiting implantation, reduces cellular metabolism to the barest minimum and stabilizes cell membrane to preserve the internal milieu in the absence of the Na^+^/K^+^ pump. Machine perfusion has been shown to be beneficial for extended-criteria donor kidneys, (22) although the results from a trial comparing machine perfusion with cold storage were equivocal (23). Although the outcome has significantly improved for both cadaver and living donor recipients, living donor recipients continue to have better long-term patient and graft survival rates. The better outcome was originally attributed to genetic matching as almost all living donors were relatives in the past; however, many recent studies have noted that living unrelated donor recipients have similar outcomes to those of non-HLA-identical living related donor recipients (10, 24). Thus, the major advantages of living donor transplants are likely due to the process itself, i.e. the ability to evaluate the donor completely, the opportunity to schedule surgery electively when both donor and recipient are in optimal condition, and the minimal ischemic time. In fact, the subset of cadaver donor recipients with excellent immediate post-transplantation graft function had similar outcomes to living donor recipients (25). The advantage of a LDKT is that it can be scheduled before dialysis is instituted. A preemptive transplant saves the recipient as well as the healthcare system the cost and complications of dialysis-access surgery and long-term dialysis (10). Living related donor represent an important potential new source of kidney grafts (26). It appears now that ABO incompatibility can be overcome with the use of immunosuppression on the basis of the results from transplantation of incompatible grafts from living related donors (27). The risk of donor mortality (28) and the possibility of coercion of donors are the major concerns with LDKT (29). However, once the procedure is explained and the willingness of living donors is established, the use of living related transplants should be as justifiable as the use of transplants from any other living related donor. Yet, efforts to increase the availability of cadaveric organs as an ultimately ideal source should not diminish. Regarding better outcomes of LDKT in comparison with well-matched DDKT (24), we found acceptable survival in both groups; although the outcome has significantly enhanced for both cadaver and living donor recipients, LDKT continues to have better long-term patient and graft survival rates.
